# Long-term tillage effect on with-in season variations in soil conditions and respiration from dryland winter wheat and soybean cropping systems

**DOI:** 10.1038/s41598-021-80979-1

**Published:** 2021-01-27

**Authors:** Diana Zapata, Nithya Rajan, Jake Mowrer, Kenneth Casey, Ronnie Schnell, Frank Hons

**Affiliations:** 1 Department of Land, Air and Water Resources, University of California, Davis, CA 95616 USA; 2 Department of Soil and Crop Sciences, Texas A&M University, College Station, TX 77843 USA; 3 Texas A&M Agrilife Research and Extension Center, Amarillo, TX 79106 USA

**Keywords:** Agroecology, Carbon cycle

## Abstract

Soil respiration from agricultural soils is a major anthropogenic source of CO_2_ to the atmosphere. With-in season emission of soil CO_2_ from croplands are affected by changes in weather, tillage, plant row spacing, and plant growth stage. Tillage involves physical turning of soils which accelerate residue decomposition and CO_2_ emission. No-tillage lacks soil disturbance and residues undergo slower decomposition at the surface. In this study, we compared with-in season soil conditions (temperature and moisture) and soil respiration from two major crops (soybean and winter wheat) by making high temporal frequency measurements using automated chambers at half-hourly intervals. The experiment lasted for 179 days. Total number of measurements made from conventional and no-tillage soybean and winter wheat plots were 6480 and 4456, respectively. Average flux after the winter-dormancy period of wheat was 37% higher in tilled soil compared to no-till soil. However, average flux during the soybean growing season was 8% lower in conventional till compared to no-till soil. This differential response of soil respiration in wheat and soybean was primarily due to tillage-induced changes in surface characteristics (residue cover) and soil environmental conditions (soil temperature and soil moisture). Results from this study can help elucidate relationships for modeling and assessment of field-scale soil CO_2_ emissions from dryland wheat and soybean crops grown in sub-tropics.

## Introduction

The atmospheric CO_2_ concentration has risen from approximately 280 ppm in 1800 to 416 ppm in 2019, largely due to industrial and agricultural development around the globe^1^. The rising CO_2_ emissions over the past two centuries have caused significant changes in the global climate, especially temperature and precipitation patterns, which manifest as environmental catastrophes such as massive floods, mega-droughts, and deadliest bushfires^2–4^. Sequestering carbon in agricultural soils can at least partially counteract the effects of anthropogenic carbon emissions^5–7^. To effectively sequester carbon in soils, carbon inputs must exceed the amounts lost through respiration.

Soil respiration (*R*_s_) or soil CO_2_ emissions is a major component of the terrestrial carbon cycle^8,9^. Globally, agricultural soils account for nearly 25% of CO_2_ released to the atmosphere from anthropogenic sources^10,11^. This efflux of CO_2_ from soils to the atmosphere includes two major respiratory fluxes: autotrophic respiration from live plant roots and heterotrophic respiration due to microbial decomposition of organic matter^12,13^. Both organic matter decomposition and root respiration are necessary processes that determine nutrient turnover and plant growth. In most natural ecosystems, carbon inputs via net primary production exceeds losses through respiration, making these systems effective terrestrial sinks of CO_2_. However, agroecosystems experience numerous management-associated events which result in major changes in the soil environment and provide the stage for rapid changes in microbial activity and CO_2_ emissions. Agriculture is also impacted by variations in climate such as drought which reduce the length of the growing season and primary production and can lead to net carbon losses. Several studies have reported croplands acting as carbon sources due to unfavorable weather and intensive management practices^14–16^.

Conventional tillage has been the most common land preparation practice used in both small and large-scale farming. Conventional tillage involves physical turning of soils and residues, which produces dramatic changes in soil physical, chemical, and biological characteristics and accelerate organic matter decomposition^17,18^. These tillage-induced changes can also result in pulses of soil CO_2_ emissions that can be several orders of magnitude greater than the typical undisturbed background rates^19^. It has been found that up to 50% of organic carbon in the top 20 cm of the soil is usually lost within three to five decades of cultivation of agricultural soils^20^.

Unlike conventional tillage, no-tillage does not involve mechanical disturbance of soils. The crop residues from the previous crop are left on the surface and the seeds are drilled directly into the soil. Previous studies have shown that no-tillage significantly reduces *R*_s_ compared to conventional methods^21–24^. However, the majority of previous studies that investigated soil CO_2_ flux from agroecosystems have been based on less frequent measurements made at weekly, bi-weekly, or monthly intervals^23,25–28^. Due to inadequacies in temporal sampling resolution, interpretations using these measurements could potentially lead to significant over- or under-estimation of soil CO_2_ emissions^29^. Besides, our understanding of diurnal cycles of *R*_s_ is still limited^30,31^. Recent advancements in automated chamber technologies have increased the number of studies that use high-frequency *R*_s_ measurements. However, most studies have been conducted in undisturbed ecosystems, with reduced anthropogenic inputs and management intensity than agricultural systems^32–34^.

Among environmental variables, soil temperature and moisture are the most important factors that determine the magnitudes of *R*_s_. Since the biological processes of respiration are temperature-dependent, soil temperature is normally the main modulator of *R*_s_ over a wide range of moisture conditions. However, since soil microorganisms require water to function, soil moisture becomes the controlling factor as soils dry out^35,36^. No-tillage leaves crop residues at the soil surface that acts as a physical barrier to evaporative water loss and slows down drying, thus providing favorable conditions for microbial activity^37^. Additionally, the slower decomposition of surface residues can provide a constant supply of carbon substrates in the upper soil layer. Therefore, measurements of temporal soil CO_2_ emissions using automated chambers across various crop growth stages, tillage, and environmental conditions can help to improve our understanding of CO_2_ emissions from agricultural soils.

In this study, the effect of long-term tillage (established in 1982) and soil environmental conditions on diurnal and with-in season soil CO_2_ emissions were investigated by collecting high temporal frequency data over a period of 179 days from two major dryland cropping systems in the U.S., winter wheat (*Triticum aestivum* L.) and soybean (*Glycine max* (L.) Merr.). Specific objectives of this study were (1) to determine the effect of tillage on with-in season soil temperature and moisture conditions in dryland wheat and soybean cropping systems, and (2) to investigate the diurnal and with-in season variations in *R*_s_ as affected by tillage and soil environmental conditions. With-in season soil CO_2_ flux from wheat was studied during the spring growth period and from soybean during early vegetative, reproductive and maturity periods. In situ measurements of high-temporal frequency respiration data from dryland wheat and soybean cropping systems are scarce. Data collected in this study can help improve the assessment of field-scale CO_2_ emissions from dryland subtropical wheat and soybean cropping systems and can assist in formulating greenhouse gas mitigation strategies.

## Results

### Effect of tillage on soil temperature and moisture

**Figure 1:**
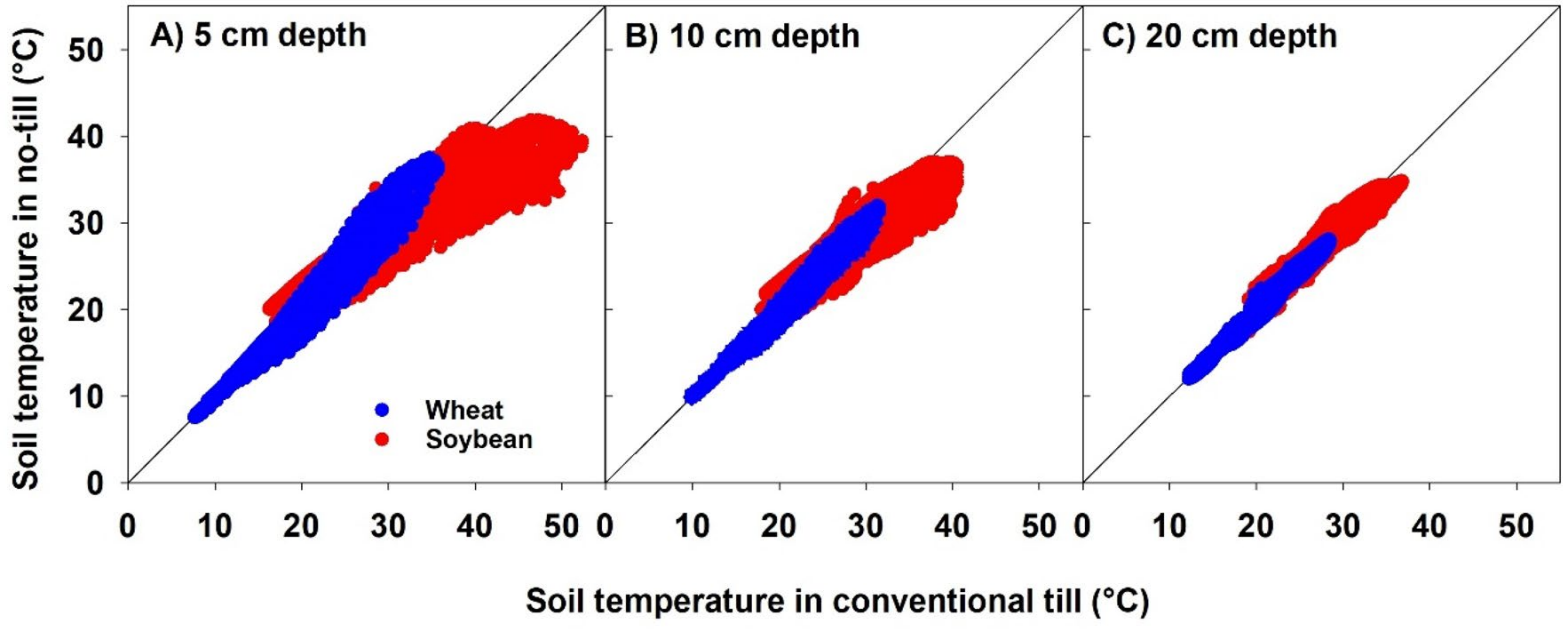
Soil temperature at **(A)** 5 cm, **(B)** 10 cm, and **(C)** 20 cm depths measured at 30-min intervals during the growing season of wheat and soybean under conventional tillage and no-tillage.

Scatter plots were constructed by plotting half-hourly records of soil temperature from tilled plots against soil temperature from no-tillage plots at three depths for both cropping systems (Fig. 1). For winter wheat, no significant difference in soil temperature with tillage was found (*P* > 0.05). The mean soil temperature in conventional tillage and no-tillage plots were 20.1 and 19.9 °C at 5 cm, 19.8 and 19.7 °C at 10 cm, and 19.7 and 19.4 °C at 20 cm depths, respectively. Unlike winter wheat, soil temperature in soybean plots were significantly different between tillage treatments at all depths (*P* < 0.0001). Tilled soybean plot had higher temperatures at all depths compared to no-tillage plot (more points below the 1:1 line in scatter plots). The mean soil temperatures in tilled and no-tillage soybean plots were 32.3 and 30.9 °C at 5 cm, 31.3 and 30.4 °C at 10 cm, and 30.3 and 29.8 °C at 20 cm depths, respectively.

**Figure 2:**
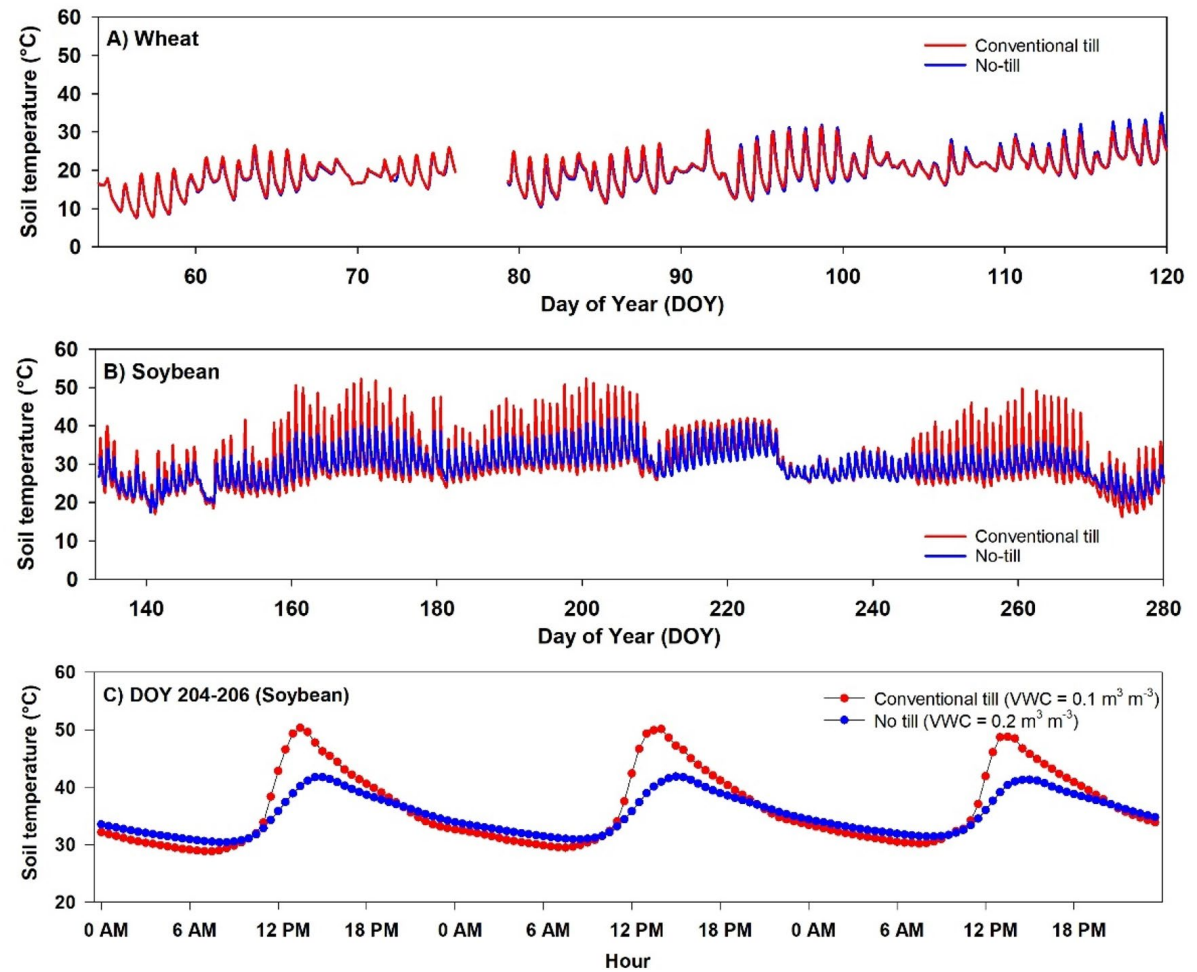
Soil temperature measured at 5 cm depth in **(A)** wheat, **(B)** soybean, and **(C)** for three consecutive days (DOY 204–206) in soybean plots under conventional tillage and no-tillage.

The diurnal fluctuations in soil temperature in tilled and no-tillage wheat plots were similar throughout the growing season at all depths (Fig. 2A). However, soils under soybean exhibited distinctive diurnal trends which were more pronounced at the 5 cm depth (Fig. 2B). The largest difference in diurnal range of soil temperature was observed when volumetric water content (VWC) was low. For example, on DOY 204–206, the maximum soil temperature at 5 cm depth in tilled soybean plot (VWC = 0.2) was at least 13 °C higher than the maximum soil temperature measured in the no-tillage plot (VWC = 0.1) (Fig. 2C). During the dry period, topsoil temperature in tilled soybean peaked earlier (1300 h) than the no-tillage treatment (1500 h). At nighttime, no-tillage soil had higher soil temperature than tilled soil on most days during the growing season (Fig. 2C).

**Figure 3:**
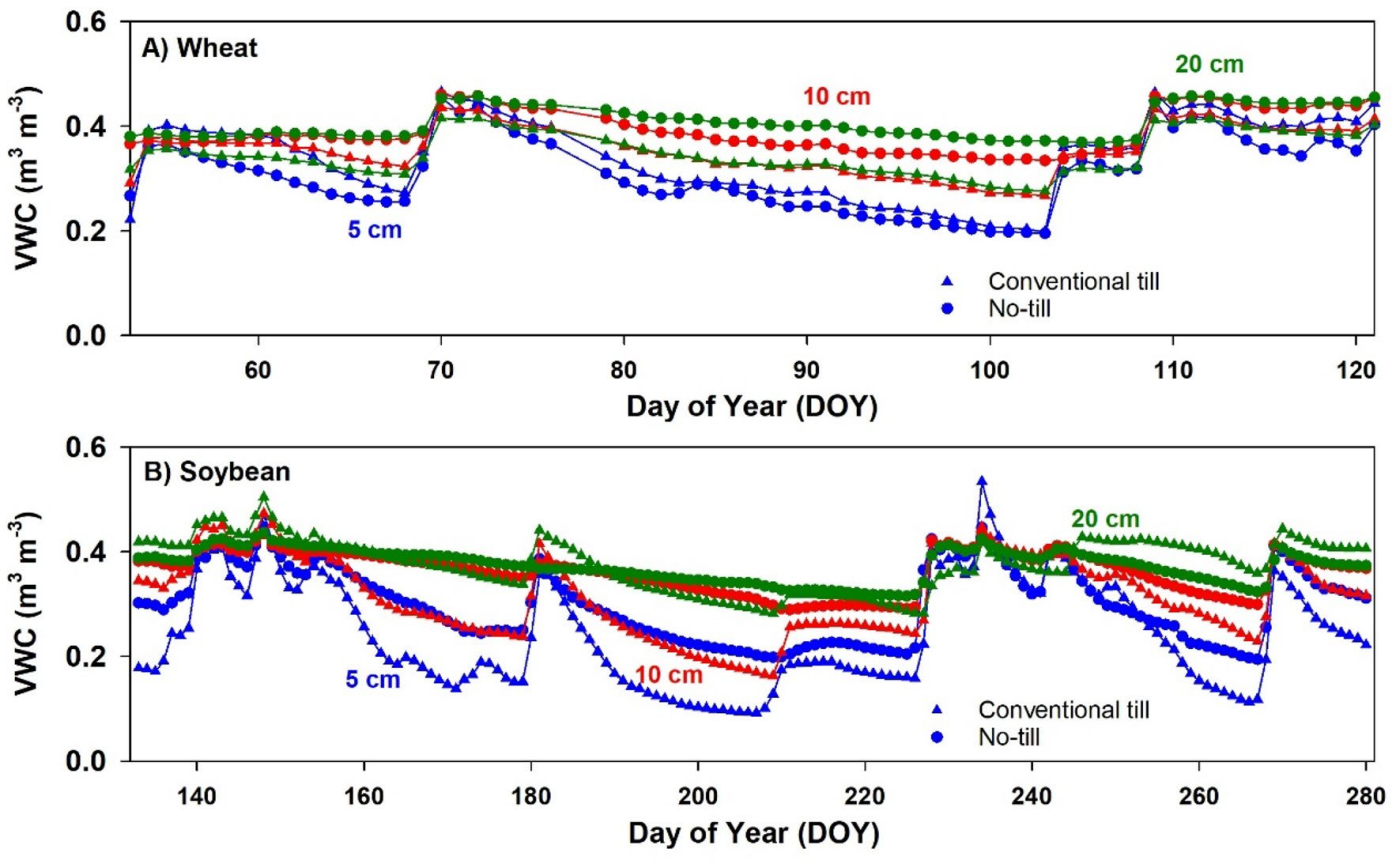
Daily volumetric soil water content (VWC) measured at 5, 10 and 20 cm depths in **(A) **wheat and **(B)** soybean under conventional tillage and no-tillage.

Daily average records of soil VWC in tilled and no-tillage plots are presented in Fig. 3A (wheat) and 3B (soybean). Soil moisture was significantly different between tillage treatments at all depths for both crops (*P* < 0.05). Except for VWC at 5 cm depth in tilled wheat, no-tillage plots had higher VWC compared to tilled plots at all depths. The difference in topsoil VWC between tillage treatments was more pronounced in soybean. On average, VWC at 5 cm depth in soybean plots was approximately 27% higher in no-tillage compared to tilled soils. The effect of tillage on VWC was most notable during drying periods, i.e., DOY 160–180, 184–208, and 252–268 in Fig. 3B. In these three periods, VWC at 5 cm depth in no-tillage was on average 48% higher than in conventional tillage.

### With-in season changes in soil respiration

**Figure 4:**
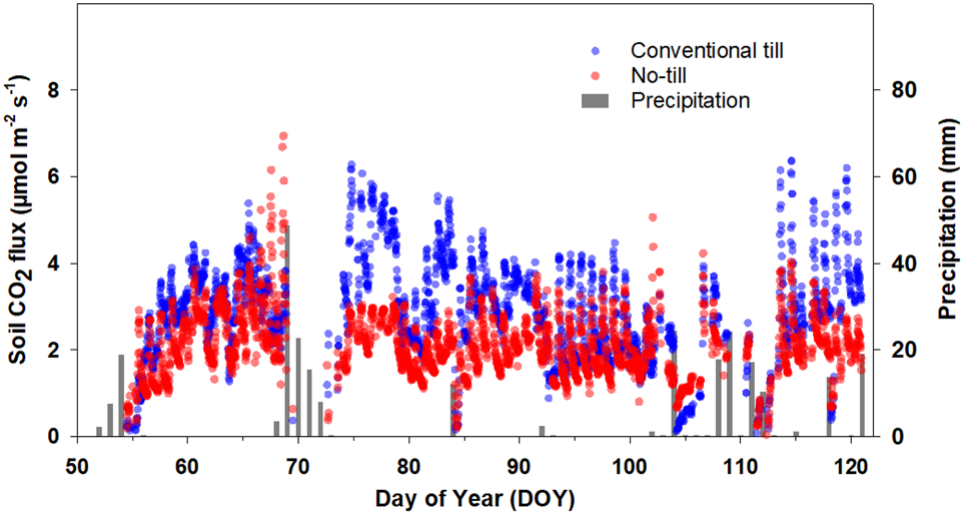
Soil CO_2_ flux measured at half-hourly intervals from wheat plots under conventional tillage and no-tillage. Total number of half-hourly measurements is 4456.

**Figure 5:**
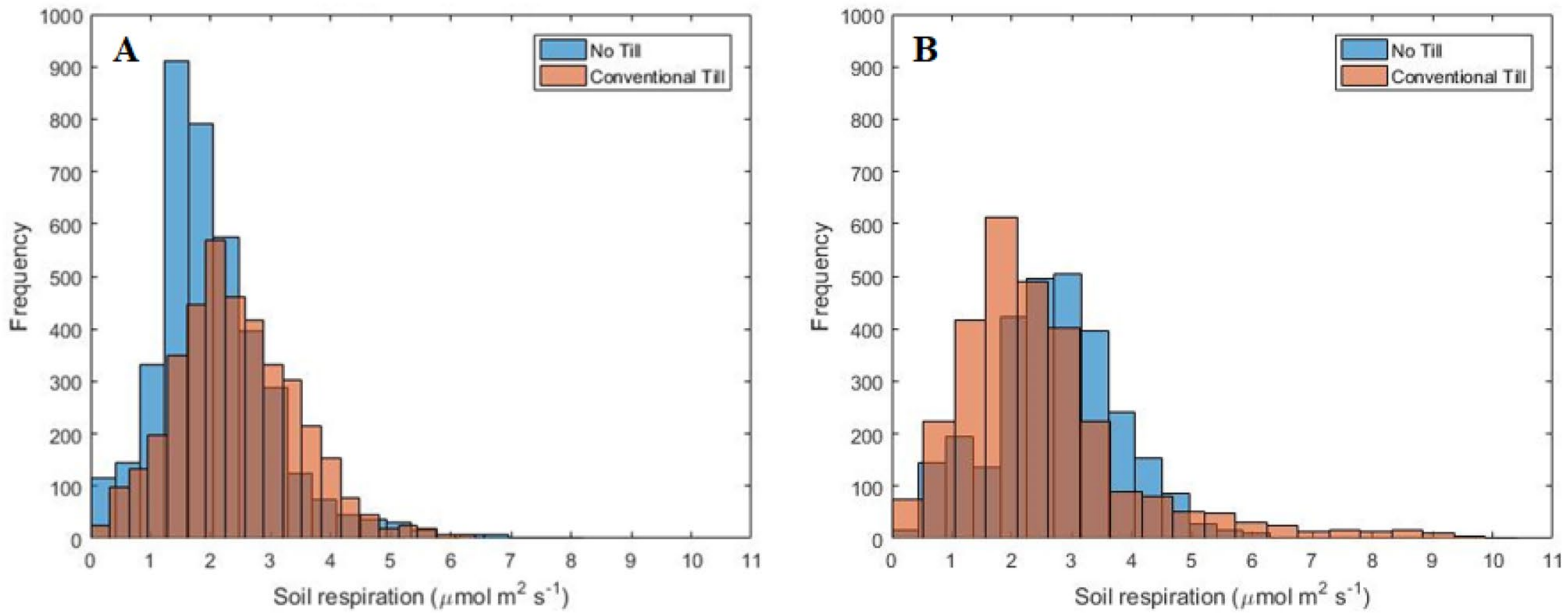
Frequency histogram of half-hourly soil CO_2_ flux data from **(A)** wheat and **(B)** soybean under conventional tillage and no-tillage.

Figure 4 presents half-hourly *R*_s_ data collected from no-tillage and tilled soils under wheat. After initial establishment and tillering in the fall, winter wheat goes through a period of dormancy from mid-December to February. During this period, vegetative growth is slow. We began measurements towards the end of the winter dormancy period and continued through the spring growth (elongation, booting, and flowering) until physiological maturity (grain filling). Half-hourly *R*_s_ values during the measurement period varied from 0.1 to 6.9 µmol m^-2^ s^-1^. Overall, *R*_s_ from tilled soil was higher than no-tilled soil except for five days immediately after the rainfall (DOY 68–72, 84, 92, 102–104, 107–112,115, 118 and 120). On average, half-hourly soil CO_2_ flux from tilled wheat soil was 37% higher (2.9 ± 1.1 µmol m^-2^ s^-1^) compared to no-tillage (2.1 ± 0.8 µmol m^-2^ s^-1^). Frequency histograms (Fig. 5) showed fluxes in no-tillage were skewed left and mainly located in a lower range compared to the conventional tillage.

**Figure 6:**
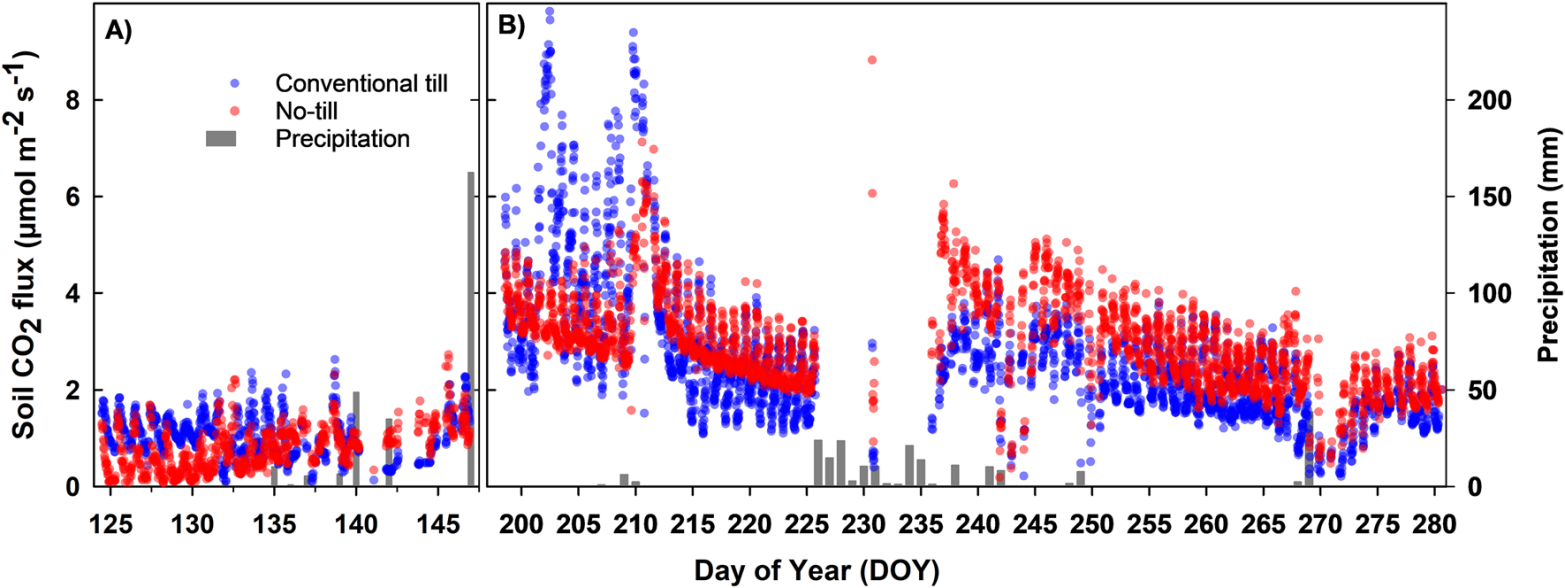
Soil CO_2_ flux measured at half-hourly intervals from soybean plots under conventional tillage and no-tillage during **(A) **vegetative and **(B)** reproductive stages. Total number of half-hourly measurements is 6480.

**Figure 7:**
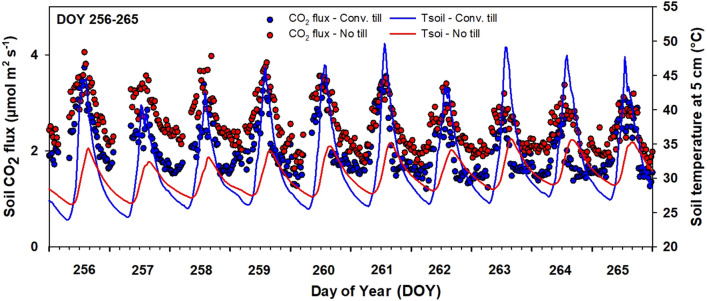
Diurnal soil CO_2_ flux and soil temperature measured at 5 cm depth in conventional tillage and no-tillage soybean during DOY 256–265.

Unlike wheat, soybean histograms (Fig. 5) showed the opposite trend with conventional tillage fluxes skewed left and distributed in a lower range. Figure 6 shows half-hourly *R*_s_ data collected from no-till and tilled soils under soybean. Half-hourly *R*_s_ values during the measurement period varied from 0.2 to 10.3 µmol m^-2^ s^-1^, and averaged 2.5 µmol m^-2^ s^-1^ in conventional tillage and 2.7 µmol m^-2^ s^-1^ in no-tillage plots. Emissions from soybean plots were the lowest during the early vegetative stage (DOY 124–146), reached maximum at the reproductive stage (DOY 198–225), and started declining during the maturity period (DOY 230–280). During the vegetative stage, daytime CO_2_ emissions from tilled soil were higher compared to no-tillage soil (*P* < 0.001). Daytime emissions from tilled soil became substantially higher during the early bloom stage (DOY 198–211). However, after a period of rainfall events (DOY 207–210), daytime CO_2_ emissions from no-tillage soil became higher than tilled soil on most days until the end of the growing season (Fig. 3). Our data also showed that nighttime soil CO_2_ emissions were always higher in no-tillage plots after the onset of the reproductive stage in soybean and a closer look at this trend is presented in Fig. 7.

## Discussion

Our results, which showed large differences in soil temperature and moisture between tilled and no-tillage treatments in soybean, are in agreement with findings from previously conducted research in similar cropping systems^38,39^. In agricultural systems, tillage is an important factor in governing the soil microclimate^40,41^. Unlike tilled systems, crop residues are not incorporated in no-tillage systems, which leads to surface accumulation of residues^17,42,43^, which then play a major role in modulating diurnal soil temperature and moisture variations. During the day, residues in no-tillage plots increase surface reflectivity and decrease heat penetration, which can reduce the available energy for evaporation, lower soil temperatures, and reduce water evaporation compared to tilled soils^44–46^. Residues may also act as a physical barrier to evaporative water loss by reducing wind effects^47^. At night, residues have the opposite effect on temperature by reducing heat loss from the soil surface and retaining heat for a longer period of time^48,49^ as it was observed in the no-tilled soybean. The soil cooling due to increased surface albedo associated with no-tillage can be comparable to that of irrigation associated changes^50^.

When crops are planted in narrow rows, such as the case of wheat in our study, canopy closure happens earlier than wider row cropping systems^51^. As high fractional vegetation cover reduces the penetration of sunlight into the canopy^52^, it also reduces heat flux into the soil^53^. Although no-tillage contributes to higher amounts of surface crop residues in both narrow and wider row cropping systems, the effect of residues on soil temperature diminishes with an increase in percent ground cover and shading of soil, which happens earlier in narrow row cropping systems^54^. This could be a possible reason for observing no significant differences in soil temperature in tilled and no-tillage wheat plots in our study.

The largest difference in the diurnal amplitude of soil temperature between tillage treatments was found in soybean. In general, tilled soils cool and warm faster than no-tilled soils^48^. Soil disturbance affects soil structure by reducing aggregate size and increasing thermal conductivity, resulting in a larger diurnal range of soil temperatures in tilled soils compared to undisturbed no-tillage soils^55^. Our results showed that no-tillage soils with residues were able to retain more water than tilled soils, which increased the soil heat capacity, causing the no-tillage soil to warm and cool slowly than tilled soils.

At our study site, *R*_s_ was generally higher in tilled wheat soil throughout the growing season. Similar results were reported in a semiarid region of the Canadian prairies with approximately 20% higher soil CO_2_ fluxes in conventional tillage wheat because of faster decomposition of surface crop residues^56^. As root respiration is a significant component of total CO_2_ emissions, other factors such as plant phenological stage^57^, secretion of root exudates^58,59^, and plant productivity^60^ also impacts the magnitude of soil CO_2_ flux. At our study site, wheat in both tillage treatments had similar growth. Hence, we assume similar contributions of autotrophic respiration to total respiration in both tillage systems. As soil temperatures and crop growth were similar in both tilled and no-tillage wheat soils, one major factor that could have caused lower soil CO_2_ flux in no-tillage at our site is soil compaction. As the soil at our no-till plots was never tilled in the past 34 years, considerable traffic from planting, spraying, and harvesting operations has led to significant soil compaction in no-tillage plots. This was evident in the higher bulk density values observed in the topsoil of no-tillage plots (1.45 g cm^−3^) compared to tilled soils (1.39 g cm^−3^). In heavy textured soils with high clay content, tillage helps to loosen up soils and increase soil macroporosity which is crucial for gas exchange^61^.

In wider-row cropping systems such as soybean, prolonged exposure of surface soil to direct sunlight and high intensity rainfall could lead to variations in soil CO_2_ emission trends as it was observed in this study. Our data revealed various trends in *R*_*s*_ during the growing season of soybean. During the early vegetative stage, soil CO_2_ emissions from tilled soils were substantially higher than from no-tillage, similar to wheat. However, towards the end of the growing season, soil CO_2_ emissions from tilled soils were lower than no-tillage soils. Two possible explanations for this include (1) availability of more labile C in the topsoil of no-tillage soil after rainfall events (DOY 207–210)^62–64^, and (2) higher soil moisture and optimum soil temperature in no-tillage soil favoring higher microbial activity and CO_2_ emissions. At nighttime, *R*_s_ was higher in no-tillage plots during most of the growing season which could be attributed to higher nighttime temperature.

## Conclusions

Among the two cropping systems studied (winter wheat and soybean), tillage induced changes in soil temperature were more prominent in soybean, a warm season crop planted in wider rows (~ 1 m). As our results indicated, tillage became less significant in modulating soil temperature in winter wheat as this crop was planted in narrow rows (~ 0.18 m), which led to early canopy closure and similar temperature profiles in both tilled and undisturbed no-tillage soils. No-tillage systems also retained more moisture than conventionally tilled soils. Tillage was a significant factor in determining soil CO_2_ emissions in both wheat and soybean cropping systems. Mixing of residues in the soil profile and more aerated conditions accelerated residue decomposition and soil CO_2_ emissions from conventional tillage soils. However, precipitaion-induced changes in soil conditions favored higher *R*_s_ in no-tillage soybean during the mid-to late-growth periods.

## Methods

### Study site

**Figure 8:**
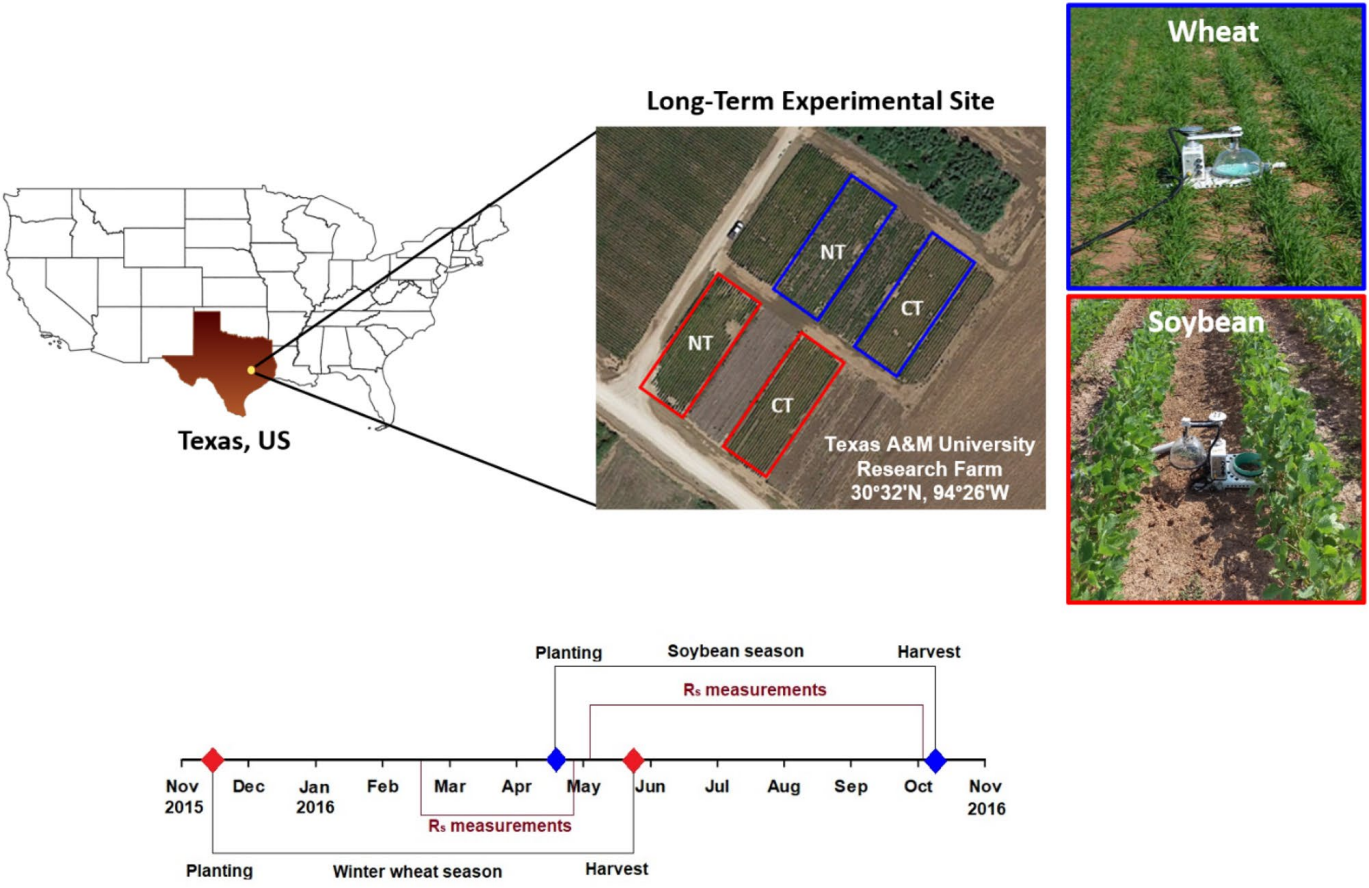
Location of the long-term agricultural experiment site at the Texas A&M University Research Farm. The conventional till (CT) and no-till (NT) plots are shown in blue (wheat) and red (soybean) boxes. A timeline is provided at the bottom that shows winter wheat and soybean growing seasons and periods of high-temporal soil respiration (*R*_s_) measurements.

The study was conducted at a long-term cropping systems field experiment site (established in 1982) at the Texas A&M University Research Farm (30°32′ N, 96°26′ W, 68.6 m a.m.s.l.) near College Station located on the Brazos River floodplain in southcentral Texas, USA (Fig. 8). Tillage treatments included conventional and no-tillage. Winter wheat plots selected for this study received nitrogen (N) fertilizer broadcasted as ammonium nitrate (68 kg N ha^-1^) in two equal split applications (after emergence and 60 days after planting). Because soybean is a legume capable of biological N fixation, fertilizer was not applied in soybean plots. Further details of this cropping systems experiment site and cultural practices can be found in^65,66^. The climate is humid subtropical with a mean annual precipitation of 1018 mm, annual average minimum air temperature of 15 °C, and maximum of 27 °C^67^. The soil at the site is classified as Weswood silty clay loam (fine-silty, mixed, superactive, thermic Udifluventic Haplustepts) with 23% sand (2–0.02 mm), 39% silt (0.02–0.002 mm), and 38% clay (< 0.002 mm) in the top 30 cm of the profile. Tillage treatments were repeated every year since the field experiment was established. Conventional tillage consisted of disking to a depth of 25 cm three to four times after harvest until next planting. No-tillage had no soil disturbance other than that resulting from planting.

### Data collection

We collected high temporal frequency soil CO_2_ flux measurements from two conventional and two no-tillage winter wheat sub-plots located at the long-term cropping systems experiment site (Fig. 8). Winter wheat was planted in 0.18 m wide rows in fall 2015 (DOY 318) and harvested in spring 2016 (DOY 146). Similar measurements were made in the soybean conventional and no-tillage plots after winter wheat. Soybean was planted in 1-m wide rows in spring 2016 (DOY 116) and harvested in late fall 2016 (DOY 280). Measurements were made using an automated soil CO_2_ flux system with long-term clear chambers that were connected to a multiplexer and an infrared gas analyzer that measured CO_2_ and water vapor concentrations simultaneously at 1 Hz frequency (Model 8100A, LI-COR Biosciences, Lincoln, NE, USA; headspace volume of 4.8 L). One chamber each was installed in conventional and no-till sub-plots. Soil chambers were programmed to measure soil CO_2_ flux sequentially from chambers at half-hourly intervals, with the actual measurement period lasting 3-min per chamber.

To facilitate soil CO_2_ flux measurements, PVC collars (20 cm diameter, height 11.4 cm, and area 317.8 cm^2^) were inserted to a depth of approximately 5 cm in between the crop rows. Measurements in the wheat plots were started on 23 February (DOY 54) and ended on 29 April (DOY 120). Measurements in the soybean plots began on 3 May (DOY 124). Data were not collected during 26 May to 16 July period due to an instrument malfunction. Measurements were resumed on 17 July and ended on 6 October (DOY 280) which coincided with the harvest of soybean. At the beginning of the measurement period, the automated closure mechanism of the soil chamber gently lowered the chamber bowl cover onto the soil collar. After the measurement was completed, the chamber moved away from the soil collar keeping the microclimate of the measured surface the same as that for the surrounding areas. Raw CO_2_ concentrations data were saved on a Secure Digital (SD) memory card onboard the infrared gas analyzer.

Soil VWC and temperature were monitored using multi-parameter water content reflectometer sensors (Model CS655, Campbell Scientific, Logan, UT, USA). Sensors were installed in both conventional and no-tillage plots at 5, 10, and 20 cm depths. Data from all CS655 sensors were measured at 30-s intervals using a CR1000 datalogger (Campbell Scientific, Logan, UT, USA) and saved as 30-min average values. Sensors were installed near the chamber in each tillage treatment. Daily air temperature and precipitation for the measurement period were obtained from a weather station located approximately 365 m from the experimental site. Soil bulk density was determined at the end of the growing season by taking 4.5 cm diameter soil cores from 0 to 30 cm depth^68^. Three soil cores were collected per plot and oven dried at 105 °C.

### Data analysis

The soil CO_2_ flux was estimated by plotting the increase in CO_2_ concentration during the measurement period against time using the SoilFluxPro software (version 4.0.5, LI-COR Biosciences, Lincoln, NE, USA). In winter wheat, a total of 4456 half-hourly soil flux measurements were made during the measurement period from conventional and no-tillage plots. The number of half-hourly measurements collected from conventional and no-tillage soybean was 6480. Each flux value was the average of measurements from the two chambers installed in each tillage treatment. This provided a high temporal frequency data set of soil CO_2_ measurements from both wheat and soybean cropping systems. Effect of soil tillage on *R*_s_ and soil environmental conditions were determined using pairwise t-tests (α = 0.05). All data processing and statistics were performed using SAS statistical software (version 9.4, SAS Institute, Cary, NC, USA).
